# Management of a Two-rooted Maxillary Central Incisor Using Cone-beam Computed Tomography: Importance of Three-dimensional Imaging

**DOI:** 10.15171/joddd.2015.037

**Published:** 2015-09-16

**Authors:** Saurabh Kumar Gupta, Payal Saxena, Shaleen Khetarpal, Mishthu Solanki

**Affiliations:** ^1^Department of Conservative Dentistry and Endodontics, Government College of Dentistry, Indore, Madhya Pradesh, India; ^2^Department of Periodontics, Government College of Dentistry, Indore, Madhya Pradesh, India; ^3^Department of Pedodontics, Modern Dental College and Research Centre, Indore, Madhya Pradesh, India

**Keywords:** Anatomic variation, cone-beam computed tomography, imaging, three-dimensional, incisor

## Abstract

We report a rare case of a two-rooted maxillary central incisor, stressing the importance of three-dimensional imaging in treatment planning and conservative approach of management. Endodontic treatment of this central incisor was carried out with a successful outcome.

## Introduction

The maxillary central incisor with more than one root is a rare condition. Most dental anatomy and endodontic texts describe the human maxillary central incisors with single root and single canal in 100% of cases,^1-3^ but a few number of dual–rooted maxillary central incisors have been reported in the literature.^4-6^

Therefore it is important that dentists be prepared to consider the existence of anatomical variations of the root canal system, even if rare, as these variations can also be found in teeth considered relatively uncomplicated for root canal treatment.

The objective of the present study is to present a rare clinical case of endodontic treatment of a maxillary central incisor with two roots and two root canals demonstrated by radiography, to emphasize the importance of cone-beam computed tomography (CBCT) examination in the treatment planning and conservative case management.

## Case Report

**Figure 1.**
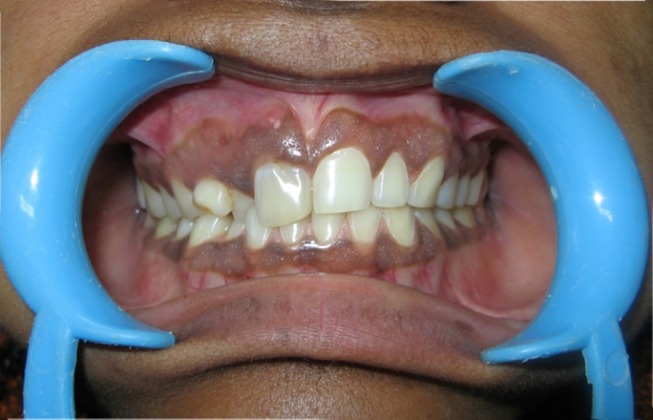
Intraoral photograph of the patient

**Figure 2.**
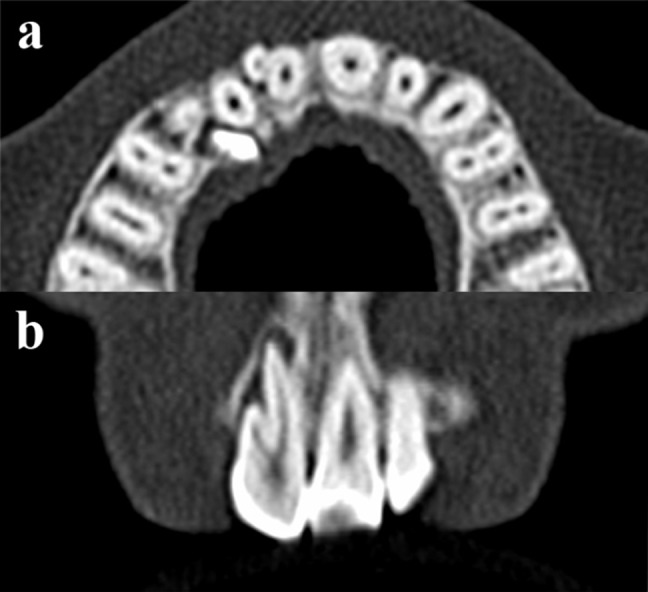
Intraoral periapical radiographs of the patient (a) shows a two-rooted maxillary right central incisor and impacted canine, (b) working length radiograph, (c) postoperative radiograph, and (d) one-year recall radiograph.

A female patient aged 32 years reported to the Department of Conservative Dentistry and Endodontics, with the chief complaint of pain in the upper anterior tooth. Examination revealed a palatally placed upper permanent right lateral incisor, buccally positioned relative to an upper retained deciduous canine tooth. The upper right central incisor was tender on percussion and presented with a facial groove with a probing depth of 1.0 mm (Figure 1). Vitality test was negative. The patient was advised a radiograph of the area in question, which showed an impacted permanent canine, and loss of lamina dura and slight amount of radiolucency in relation to the periapical area of the upper right central incisor (Figure 2a). There was an additional root present with the central incisor. Since the deciduous tooth was firm, root canal treatment of the central incisor, extraction of impacted canine and orthodontic correction of palatally placed lateral incisor was advised but the patient refused to undergo any surgical treatment. A CBCT was advised to rule out the association of the impacted canine with the central incisor and to identify the exact position of the additional root of central incisor.

**Figure 3.**
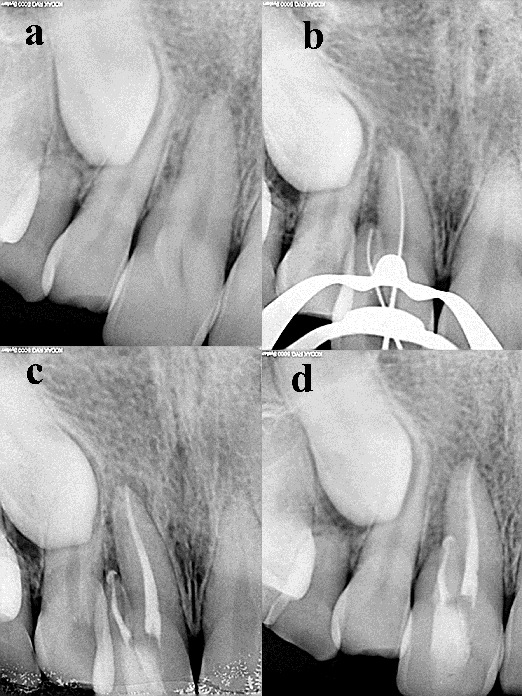
CBCT images of the patient; (a) shows the coronal section of the two-rooted maxillary right central incisor; (b) is the axial section.

The CBCT ruled out the association of impacted canine with the central incisor (Figure 3a) and showed a periapical lesion related to both the roots of the central incisor (Figure 2b). Contrary to our expectation in relation to the additional root being positioned in the middle of the labiolingual length of the tooth, we found it to be positioned more facially (Figure 1a). A three dimensional image of the tooth is presented in Figure (a and b. A nonsurgical endodontic therapy was decided after discussion with the patient.

**Figure 4.**
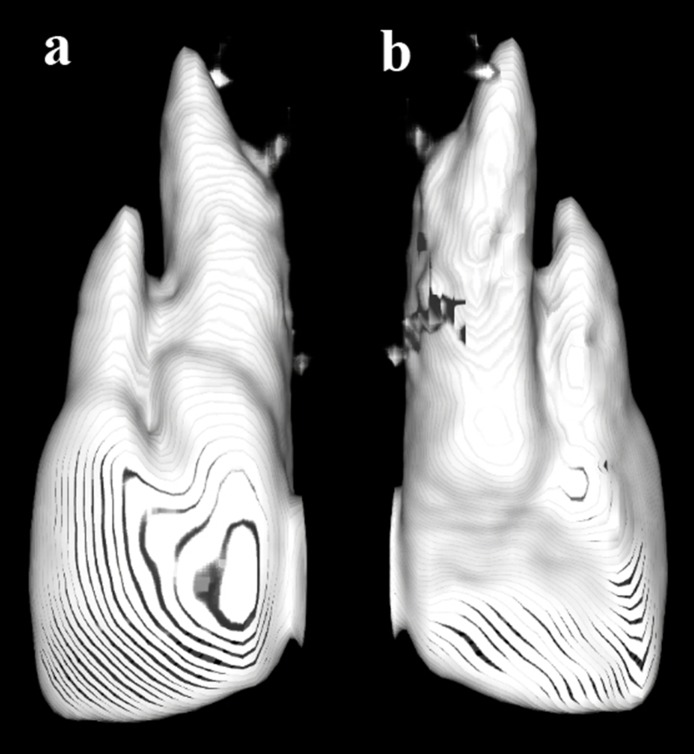
Three-dimensional image of maxillary right central incisor: (a) facial view, (b) palatal view.

After rubber dam application, endodontic access was gained. Two canal orifices (mesial and distal) were located with an endodontic explorer (DG-16, Dentsply Instruments, Surrey, UK). The working length of both canals was determined radiographically (Figure 2b) and confirmed with an apex locator (Propex II, Dentsply international, Surrey, UK). The root canals were cleaned and shaped using rotary Protaper files (Dentsply International, Surrey, UK). The mesial canal was instrumented to #F4 and distal canal to #F3. The root canals were copiously irrigated with 2.5% sodium hypochlorite solution. Calcium hydroxide/distilled water paste was then placed as an intracanal medicament. After 2 weeks, the tooth was asymptomatic and the root canals were obturated with corresponding Protaper gutta-percha and AH Plus sealer (Dentsply De Trey, Germany) (Figure 2c). During sealing of the endodontic access cavity, the patient was given the option of restoring the facial groove with composite resin, which she refused. One-year follow-up radiograph presented satisfactory healing (Figure 2d). The patient was absolutely asymptomatic and satisfied with the treatment outcome.

## Discussion

The internal anatomy of the maxillary central incisor is usually considered well known and presence of more than one root is a rare condition. Unexpected morphological variation makes endodontic treatment a challenge for dentists. Clinicians should be aware of all the possible root canal anatomical configurations and different diagnostic resources which lead to a successful endodontic treatment.^7^

Radiographic examination is an essential component in the management of endodontic problems. It is involved in all aspects of endodontic treatment from diagnosis and treatment planning to assessing the outcome. The amount of information gained from conventional films and digitally captured periapical radiographs is limited by two-dimensional representation of three-dimensional (3D) anatomy. As a result of superimposition, periapical radiographs reveal limited aspects of the 3D anatomy. In addition, there might also be geometric distortion of the anatomic structures being imaged.^8^ These problems can be addressed by the use of CBCT, which can produce 3D images of individual teeth and aid in better understanding of the root canal morphology. Ruling out CBCT in our present case would have led to over-extension of our endodontic access preparation and might have resulted in gouging and even perforation. In addition, the CBCT assisted us in ruling out the association between the impacted canine and the central incisor, confirming the correct spatial position of the two roots and locating the canal orifices accurately, all of which helped us in adopting a conservative approach to the present problem. CBCT has a number of potential advantages compared with conventional CT, including a decrease in the size of the irradiated area by collimation of the primary x-ray beam, a significant reduction in the effective dose of radiation, image accuracy, rapid scan time and reduced image artifacts.^9^ A case of two-rooted maxillary incisor was reported where anatomical complexity of the tooth led to perforation in the distal root during conventional treatment, thus a surgical approach was additionally required.^10^

The diagnosis of any other shape abnormality as germination or fusion was excluded, since according to Shäfer,^11^ in case of germination, the tooth structure is usually one with two completely or incompletely separated crowns that have one root and root canal while in fusion, depending upon the stage of development of teeth at the time of union, a single large tooth with complete fusion or only the fusion of roots might occur. The dentin is always confluent in true fusion and might have fused or separated root canals. Since the present case does not conform to the definitions of germination or fusion and to keep it simple, avoiding any ambiguity, it was depicted as a two-rooted maxillary central incisor.

It has been observed that the anomalies related to the number and distribution of roots and root canals have a tendency of occurring bilaterally, and the rarer the aberration, the more likely it is expected to be bilateral,^12^ but in the present case the two-rooted central incisor was present on the right side only.

## Conclusion

The present case demonstrates the variability of root and root canal morphology in teeth and one should be vigilant even in treating apparently routine cases as one might encounter a deviation from the usual. In addition, the importance of three-dimensional imaging is stressed. Proper coordination between imaging and treatment plan leads to a more conservative approach with predictable results.
